# Properties of oscillatory neuronal activity in the basal ganglia and thalamus in patients with Parkinson’s disease

**DOI:** 10.1186/s40035-018-0123-y

**Published:** 2018-07-25

**Authors:** G. Du, P. Zhuang, M. Hallett, Y.-Q. Zhang, J.-Y. Li, Y.-J. Li

**Affiliations:** 1 Beijing Institute of Functional Neurosurgery, Xuanwu Hospital, Capital Medical University, No.45 Changchun Street , Xicheng District, Beijing, 100053 China; 2 Human Motor Control Section, Medical Neurology Branch, NINDS, NIH, Bethesda, MD USA; 3 Center of Parkinson’s disease, Beijing Institute for Brain Disorders, Beijing, China; 4 Key Laboratory of Neurodegenerative Diseases (Capital Medical University), Ministry of Education, Beijing, China

**Keywords:** Parkinson’s disease, Basal ganglia, The subthalamic nucleus, The globus pallidus internus, The ventrolateral thalamus, Oscillatory activity, Microelectrode recordings

## Abstract

**Background:**

The cardinal features of Parkinson’s disease (PD) are bradykinesia, rigidity and rest tremor. Abnormal activity in the basal ganglia is predicted to underlie the mechanism of motor symptoms. This study aims to characterize properties of oscillatory activity in the basal ganglia and motor thalamus in patients with PD.

**Methods:**

Twenty-nine patients with PD who underwent bilateral or unilateral electrode implantation for subthalamic nucleus (STN) DBS (*n* = 11), unilateral pallidotomy (*n* = 9) and unilateral thalamotomy (n = 9) were studied. Microelectrode recordings in the STN, globus pallidus internus (GPi) and ventral oral posterior/ventral intermediate of thalamus (Vop/Vim) were performed. Electromyography of the contralateral limbs was recorded. Single unit characteristics including interspike intervals were analyzed. Spectral and coherence analyses were assessed. Mean spontaneous firing rate (MSFR) of neurons was calculated. Analysis of variance and X^2^ test were performed.

**Results:**

Of 76 STN neurons, 39.5% were 4–6 Hz band oscillatory neurons and 28.9% were β frequency band (βFB) oscillatory neurons. The MSFR was 44.2 ± 7.6 Hz. Of 62 GPi neurons, 37.1% were 4–6 Hz band oscillatory neurons and 27.4% were βFB neurons. The MSFR was 80.9 ± 9.6 Hz. Of 44 Vop neurons, 65.9% were 4–6 Hz band oscillatory neurons and 9% were βFB neurons. The MSFR was 24.4 ± 4.2 Hz. Of 30 Vim oscillatory neurons, 70% were 4–6 Hz band oscillatory neurons and 13.3% were βFB neurons. The MSFR was 30.3 ± 3.6 Hz. Further analysis indicated that proportion of βFB oscillatory neurons in STN and GPi was higher than that of similar neurons in the Vop and Vim (*P* < 0.05). Conversely, the proportion of 4–6 Hz band oscillatory neurons and tremor related neurons in the Vim and Vop was higher than that of STN and GPi (*P* < 0.05). The highest MSFR was for GPi oscillatory neurons whereas the lowest MSFR was for Vop oscillatory neurons (*P* < 0.005).

**Conclusion:**

The alterations in neuronal activity in basal ganglia play a critical role in generation of parkinsonism. β oscillatory activity is more prominent in basal ganglia than in thalamus suggesting that the activity likely results from dopaminergic depletion. While both basal ganglia and thalamus have tremor activity, the thalamus appears to play a more important role in tremor production, and basal ganglia β oscillatory activity might be the trigger.

## Background

Parkinson’s disease (PD) is a progressive neurological degenerative disorder characterized by a severe loss of dopaminergic neurons in the substantia nigra pars compacta. The cardinal features of PD are motor symptoms, including slowness of movement (bradykinesia), akinesia, rigidity and tremor at rest [1]. Abnormal activity in the basal ganglia neurons is predicted to underlie the mechanism of parkinsonian symptoms [2]. The altered neuronal activity can be observed in the human basal ganglia because of the stereotactic surgery for PD that provides the unique opportunity to record from these regions in relation to parkinsonian motor deficits.

Classical models of basal ganglia function are based on discharge rates and patterns in the basal ganglia structures and predict those in PD. The model postulates that striatal dopamine depletion results in an increased firing rates in the inhibitory striatal output neurons of the indirect pathway, leading to the inhibition of the external segment of globus pallidus (GPe) and subsequent disinhibition of the subthalamic nucleus (STN) and reinforcing the inhibitory the internal segment of globus pallidus (GPi)/the substantial nigra reticularis (SNr). The loss of dopaminergic facilitation of the direct inhibitory pathway likely further increases the GPi/SNr activity. Increased basal ganglia output from GPi/SNr to the thalamus was considered to responsible for excessively inhibiting thalamocortical interactions, and, thus, reducing excitability of cortical neurons [3, 4]. The increased neuronal firing rate in the GPi and STN has been found in animal model of PD [3–9] and are supported by microelectrode recording studies in PD patients undergoing surgery for symptoms [10, 11]. These studies indicate that neuronal activities in the basal ganglia neurons are increased compared to normal. In accordance with the model, lesions or stimulation in GPi and STN of PD patients improve parkinsonian symptoms [12, 13]. Administration of dopaminergic medication in 1-methyl-4-phenyl-1,2,3,6-tetrahydropyridine (MPTP) primate model of PD and patients with PD decreased GPi and increased GPe neuronal activity are consistent with the prediction of the model [14, 15]. Moreover, reduced firing rates of neurons in the ventral oral anterior of thalamus (Voa)/the ventral oral posterior of thalamus (Vop), the pallidal receiving area of the thalamus was also obtained from patients of PD [16, 17]. However, the downstream effects of abnormal basal ganglia output on firing rate in the thalamus have been studied to a limited extent and comparative studies of neuronal activity in the basal ganglia receiving regions of thalamus have been inconsistent [18–21]. While some studies show decreased neuronal firing [18, 19], some studies found no firing rate change [20] and others show increase in firing rates [22].

Alternatively, the widespread changes in the firing patterns of basal ganglia neuron have been proposed to be key pathophysiological mechanism in PD. In PD, the incidence of neuronal bursting activity in the GPi and STN is increased [6, 8]. These studies showed that there were neurons with periodic bursting neuronal activity at tremor frequency (4–6 Hz) in the STN [23], GPi [24] and the ventral thalamus [25]. These tremor frequency oscillatory neurons are frequently correlated with muscular activity of limb tremor [26, 27]. The tremor frequency oscillatory activity is not only observed in basal ganglia nuclei but also observed in the pallidal receiving area, Vop, and the ventral intermediate of thalamus (Vim) [26–28]. The Vim is well known excellent target to relieve parkinsonian tremor, however, the region primarily receives input from cerebellum.

Further, microelectrode recording studies showed that there were neurons with periodic intermittent bursts at β frequency range (10–30 Hz) oscillation in STN [23, 27] in PD patients. These studies demonstrated that there is a relationship between β oscillatory activity in the STN and the parkinsonian akinetic/rigid state [29, 30]. In PD patients, β activity in the STN and GPi is the principal activity in the local field potential (LFP) from the macroelectrodes used for therapeutic high frequency stimulation of these regions [31–34]. These LFPs exhibit oscillations in tremor frequency range and β frequency ranges and correlate with single units [35, 36]. The β oscillatory discharge and LFPs observed in PD patients are rapidly reversed by treatment with dopaminergic medication [37, 38]; the reduction of β oscillations is associated with the degree of improvement in akinesia/rigidity following dopamine-replacement therapy [37, 39], and are positively correlated with the patients’ response to the medication [38, 39], suggesting that the synchronized oscillatory activity might result from dopamine deficiency in basal ganglia [37–39]. More recent studies showed that the proportion of different patterns of oscillatory neurons seems to associate with different phenotype in PD [30, 40]. However, the relationship between pattern of oscillatory neurons and phenotypes remains to be explored.

Despite the abundance of evidence suggesting that changes in firing rate and oscillatory patterns in basal ganglia and ventral thalamic neurons are associated with parkinsonian symptoms, the pathophysiology of PD is still unclear. In the current study, we take the advantage of microelectrode targeting of basal ganglia nuclei STN and GPi as well as the Vop/Vim of thalamus during surgery treatment for PD and attempt to further examine neuronal firing rate and patterns of oscillatory neurons of these structures. We expect to provide useful data to further understand the mechanisms of PD.

## Methods

### Patients

Twenty-nine patients with PD (15 males, 14 females; age: 59.9 ± 6.9 years) who underwent bilateral or unilateral electrode implantation for STN DBS (*n* = 11), unilateral pallidotomy (*n* = 9) and unilateral thalamotomy (*n* = 9) were studied. The diagnoses of PD were based on medical history, physical examinations, L-dopa response; laboratory tests and MRI scans to exclude other diseases. The mean duration of disease was 6.1 ± 3.2 years; mean dosage of L-dopa was 562.1 ± 329.0 mg/daily. The mean Unified Parkinson’s Disease Rating Scale (UPDRS) III (Motor) score was 45.5 ± 11.4 during “off” medication, and the mean Hoehn and Yahr score was 2.6 ± 0.70 at the time of surgery. Based on the assessment of UPDRS III subscores and clinical features, patients with mixed rigidity, bradykinesia and tremor are selected for STN DBS; patients with rigidity, tremor and presented L-dopa induced dyskinesia are selected for unilateral pallidotomy; patients with prominent and disabling tremor are selected for unilateral thalamotomy.

The details of demographic and clinical characteristics of the patients are presented in Table 1.

**Table 1:** The demographic details of the patients

Patient	Age/ gender	Disease duration (years)	Motor UPDRS OFF/ON	Hoehn Yahr Score	L-dopa equivalent doses (mg/day)	Side of micro- electrode tract of nuclei	No. of neuron analyzed		
							4–5 Hz band oscillatory neuron(%)	βFB oscillatory neuron (%)	non-oscillatory neuron (%)
1	46/M	2	63.5/35.5	2.5	600	B-STN	2(28.6)	2(28.6)	3(42.9)
2	57/F	4	40/16.5	2.5	600	B-STN	6(85.7)	1(14.3)	0(0)
3	67/M	7	62.5/43	4	775	B-STN	6(54.5)	3(27.3)	2(18.2)
4	56/M	8	20/12	1.5	750	L-STN	3(42.9)	2(28.6)	2(28.6)
5	62/F	10	41.5/21	3	150	R-STN	4(80)	0(0)	1(20)
6	71/F	10	40/19	3	675	L-STN	2(33.3)	2(33.3)	2(33.3)
7	56/M	5	45/25.5	2.5	250	L-STN	3(37.5)	1(12.5)	4(50)
8	64/F	12	57.5/40	3	375	R-STN	4(50)	0(0)	4(50)
9	67/M	4	30/18.5	2.5	400	L(STN)	0(0)	2(50)	2(50)
10	66/F	7	61.5/31	3	600	B-STN	0(0)	9(90)	1(10)
11	68/M	4	52/26.5	2.5	675	B-STN	0(0)	0(0)	3(100)
12	61/F	5	44.5/24.5	2	300	L-GPi	1(14.3)	2(28.6)	4(57.1)
13	59/F	6	42.5/25.5	2.5	600	L-GPi	4(44.4)	2(22.2)	3(33.3)
14	57/M	6	56.5/32	3	600	R-GPi	8(66.7)	2(16.7)	2(16.7)
15	62/F	5	60/37.5	2.5	150	R-GPi	2(33.3)	2(33.3)	2(33.3)
16	51/F	7	52.5/26.5	2.5	400	L-GPi	3(30)	2(20)	5(50)
17	48/M	5	32.5/18	2.5	300	R-GPi	4(66.7)	1(16.7)	1(16.7)
18	58/M	10	59.5/33.5	4	600	L-GPi	1(20)	2(40)	2(40)
19	61/M	8	42/23.5	2.5	1200	L-GPi	0(0)	1(50)	1(50)
20	56/F	6	49/28	3	1800	R-GPi	0(0)	3(60)	2(40)
21	61/M	1	35/17.5	1.5	300	L-Vop/Vim	4(40)/3(30)	1(10)/1(10)	1(10)/0(0)
22	68/M	5	30/15.5	1.5	200	L-Vop/Vim	5(50)/0(0)	0(0)/0(0)	3(30)/2(20)
23	67/F	15	53/33	3	800	L-Vop/Vim	3(50)/3(50)	0(0)/0(0)	0(0)/0(0)
24	49/M	6	55/32	2.5	600	R-Vop/Vim	4(44.4)/4(44.4)	0(0)/0(0)	1(11.1)/0(0)
25	70/M	3	33/20	1.5	400	R-Vop/Vim	6(54.5)/1(9.1)	1(9.1)/1(9.1)	1(9.1)/1(9.1)
26	64/F	2	39.5/27.5	2.5	600	R-Vop/Vim	0(0)/2(100)	0(0)/0(0)	0(0)/0(0)
27	56/F	9	46.5/28.5	4	400	L-Vop/Vim	5(50)/3(30)	0(0)/1(10)	0(0)/1(10)
28	49/F	2	37.5/20.5	1.5	600	L-Vop/Vim	2(18.2)/5(45.5)	2(18.2)/1(9.1)	1(9.1)/0(0)
29	61/M	3	36.5/24	2.5	600	R-Vop/Vim	0(0)/0(0)	0(0)/0(0)	4(80)/1(20)

Patients 1–11 belong to STN group, patients 12–20 belong to GPi group, patients 21–29 belong to Vop/Vim group; M, male; F, female; UPDRS, United Parkinson’s Disease Rating Scale; L-dopa equivalent daily doses (mg), calculated as sum of the dose of regular levodopa-benserazide or levodopa-carbidopa; B, Bilateral; L, Left; R, Right; No: number; βFB: β frequency band oscillatory neurons; STN: the subthalamic nucleus; GPi: the globus pallidus internus; Vop: the ventral oral posterior nucleus of thalamus; Vim: the ventral intermediate of thalamus

This study was approved by the Ethics Committee of Xuanwu Hospital, Capital Medical University. All patients signed written informed consent.

### Surgical procedure and electrophysiology

The methods are described in detail in previous studies [29, 41]. A sagittal magnetic resonance image (Siemens1.5 Tesla, Sonata, Germany) using the Cosman-Roberts-Wells (CRW) frame (Radionics, Burlington, MA, USA) was obtained prior to the stereotactic surgery. The targets of the STN, GPi and VL were calculated based on a human atlas. The coordinates of the target point of the STN were: 12 mm lateral, 1 mm posterior, and 4 mm inferior to the midcommissural point. The anteroposterior and lateromedial angles were 60°and 12, respectively. In the present study, coordinates of the target of the GPi were 2 mm anterior to the midpoint of the AC-PC line, 4–6 mm below the AC-PC line, and 18–22 mm lateral to the midline. The coordinates to the target of the Vop/Vim were 4 to 8 mm anterior to the PC, 12 to 15 mm lateral to the midline, and 0 to 2 mm superior to the AC-PC line.

Microelectrode recordings in the STN, GPi and the Vop/Vim were performed during targeting. A tungsten microelectrode with tip size 10–20 μm and resistances from 0.1 to 0.5 MΩ at 1000 Hz (Alpha Omega Engineering, Nazareth, Israel) was used. The all microelectrode started 10 mm from the target and advanced in 0.5-mm increments.

For the STN, the target was identified based on: when the electrode entered the dorsal border of the STN, there was increased background and high-frequency activity with relatively irregular neuronal discharges as well as burst activity. As the electrodes were advanced past the ventral border of the STN, background noise gradually decreased until the substantia nigra pars reticulata was reached. This was identified by a higher frequency, which became more regular and had lower amplitude discharges compared with the STN.

For the localization of GPi, the final target was identified according to the landmarks which included the location of the optic tract (OT) and internal capsule as identified by using strobe light and macrostimulation. The presence of ‘border neurons’ marking the boundaries of nuclear segments with the relative ‘quiet’ white matter laminae. The characteristic discharge patterns of neurons in GPi (including the external (GPie) and internal (GPii) of GPi)) was based on (1) the stereotactic location of the tract, corrected for depth based on the level of the dorsal border of the optic tract (determined by a macrostimulation threshold for visual sensation); (2) on the basis of degree of multi-unit (cell-dense versus cell-sparse zones); (3) the amount of background noise in the recordings, and (4) the location of border neurons. Besides the border cells and silent zone in white lamina, the typically highly active neuronal discharges in the GPi were identified.

For the Vop/Vim, the final targets were confirmed by functional properties of thalamic cells. First, the electrode trajectory was directed toward the principal somatic sensory nucleus (the ventral caudal, Vc) of thalamus where the majority of cells responded to cutaneous stimulation. Therefore, Vc, the physiologically defined region, could be used as the reliable landmark for the localization of Vop and Vim [42]. The method was used to identify the Vop neurons: neurons located ≥ 3 mm anterior to the tactile border between the Vim and the Vc, which based on the stereotactic atlas of Schatenbrand and Wharen [43], and the angle of recording trajectory in the present study is approximately the border between the Vim and Vc. Neurons were identified as being in Vim if they were located within 3 mm anterior to the tactile border between the Vim and the Vc [16]. Based on the definition, the length of Vop neurons recorded approximately 7 mm whereas the length of Vim neurons recorded approximately 3 mm.

In the present study, all isolated units were monitored for periods of between 15 s to several minutes to study spontaneous firing rates and neuronal oscillatory activity.

Recorded signals from the microelectrode were amplified (× 20,000) and filtered (with bandpass of 200 Hz–10 kHz). The signals were sampled at 12 kHz. Three channels of electromyograms (EMGs) were simultaneously recorded using surface electrodes from the extensor carpi radialis (ECR), flexor carpi radialis (FCR), and the tibialis anterior (TA) muscles on the contralateral limbs. EMG signals were amplified and sampled at 3 kHz. All recordings were obtained with patients at rest using the MicroGuide system (AlphaOmega Engineering, Nazareth, Israel).

Patients were withdrawn from medications overnight before surgery and were awake during the entire operation to ensure cooperation.

### Data analysis

Neuronal and EMG signals were converted into Spike2 format (Cambridge Electronic Design, Cambridge, UK) for discrimination [23, 27]. Only stable, well-isolated single neurons seen in recordings longer than 15 s without voluntary movements and artifact were processed. Spikes with a signal-to-noise ratio greater than 2:1 were used. The interspike interval (ISI), the ISI histogram, and the coefficient of variation (CV) of ISI were performed to explore the mean spontaneous firing rate (MSFR) and patterns.

All neuronal and EMG signals were then full wave rectified and imported into MATLAB 7 (The MathWorks, Natick, MA, USA). Power spectrum density (PSD) analysis evaluated neuronal oscillation. A Hanning window at a 50% overlap between windows was used. The significant oscillatory frequencies were determined when exceeding a threshold of 5 SD above the mean power in the 30–100 Hz band [23].

The relationship between neuronal oscillation and EMG was determined using coherence analysis. A coherence of > 0.42 at a given frequency indicated that the two signals were likely to be related linearly at that frequency (*p < 0.05*) [22]. All data analysis was carried out using Spike II 7.02 (Cambridge Electronic Design, Cambridge, UK), MATLAB 7.0 (The MathWorks, Natick, MA, USA) and Origin 7.5 (OriginLab Corporation, Northampton, MA, USA).

### Statistical analysis

All data were expressed as mean ± standard deviation. Neuronal firing rate of different types of neuron within and between the nucleus groups was statistically analyzed using a one way analysis of variance (ANOVA) and Bonferroni multiple comparison post hoc test, with a level of significance of α = 0.05. A comparison of different types of oscillatory neuronal patterns by the nucleus groups was performed using X^2^ analysis with the null hypothesis that the proportions were the same. Statistical significance was set at *p* < 0.05.

SPSS 17.0 (SPSS, Chicago, IL, USA) and Origin 7.5 (OriginLab Corporation, Northampton, MA, USA) software were used for statistical analysis.

## Results

A total of 212 neurons were analyzed in 29 PD patients. Of these neurons, 76 neurons were identified from 16 STNs, 62 neurons were identified from 9 GPis and 74 neurons were identified from 9 Vop/Vims (44 Vop neurons and 30 Vim neurons). The length of the mean durations of neuronal recordings showed no significant difference among four nuclei: 38.4 ± 24.2 s for STN; 27.6 ± 22.8 s for GPi, and 36.4 ± 27.8 s for Vop/Vim (*P* > 0.05). In this report, the main analysis focuses on oscillatory neurons.

### Subthalamic oscillatory neurons

Of 76 STN neurons, 30 (39.5%) were 4–6 Hz band oscillatory neurons at tremor frequency 4.6 ± 0.4 Hz (range 4–5.5 Hz); Of these 4–6 Hz band oscillatory neurons, 12 (40.0%) were significantly coherent with limb tremor (coherent coefficient at range: 0.45–0.85, mean: 0.68 ± 0.12), defined as tremor related oscillatory neurons; (2) 22 (28.9%) were β frequency band (β FB) oscillatory neurons with intermittent, periodic bursting at frequency of 20.8 ± 6.3 Hz (range 10–30 Hz); (3) 24 (31.6%) were non-oscillatory neurons with irregular activity showing no oscillation, termed non-oscillatory neuron. Further ISI analysis showed that the MSFR of 76 neurons were 39.8 ± 9.9 Hz (range18.1–67.8 Hz) and 52 oscillatory neurons was 44.2 ± 7.6 Hz (range 30.5–67.8 Hz) (*P* = 0.06).

**Fig. 1:**
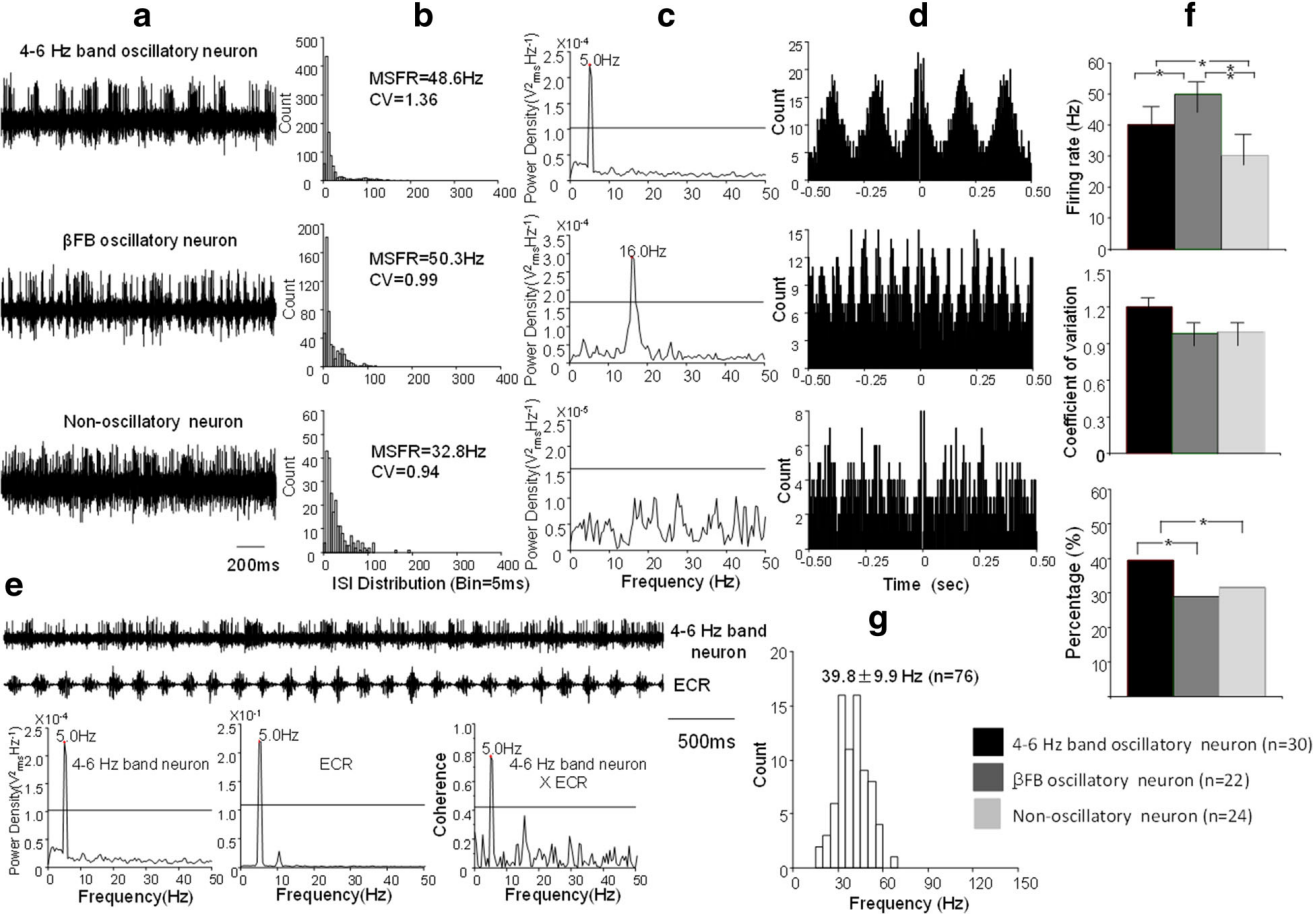
Characteristics of 4–6 Hz band, *β*FB, and non-oscillatory neurons in the STN. **a** Patterns of three neurons with 4–6 Hz band, *β*FB, and non-oscillation; **b** ISI histograms of the three neurons and their MSFR of 48.6 Hz, 50.3 Hz and 32.8 Hz; **c** Power density spectrum of the three neurons at peak power of 5 Hz, 16 Hz, and no power; the horizontal line indicates a significant oscillatory level of the power of spike train signal; **d** autocorrelation histograms of three patterns of oscillatory neuronal activity; **e** An example of STN 4–6 Hz band oscillatory neurons and its coherence with limb tremor. The top trace shows raw data of a neuron with 4–6 Hz band oscillation that corresponds to the limb tremor (FCR); below shows spectral analysis of 4–6 Hz band oscillatory neuron, limb tremor of FCR, and 4–6 Hz band neuron x FCR which is the coherence between them; Horizontal line indicates a significant coherent level at 0.42 (*P* < 0.05). **f** Comparisons of MSFR, CV and percentages of4–6 Hz band, *β*FB oscillatory neurons and non-oscillatory neurons; **g** Histogram demonstrates distribution of MSFR of total STN neurons

Fig. 1 demonstrates examples of 4–6 Hz band and β frequency band oscillatory neurons and non-oscillatory neurons representative for most subthalamic neurons. The firing rate and CV and proportion of three types of neurons were compared.

### GPi oscillatory neurons

**Fig. 2:**
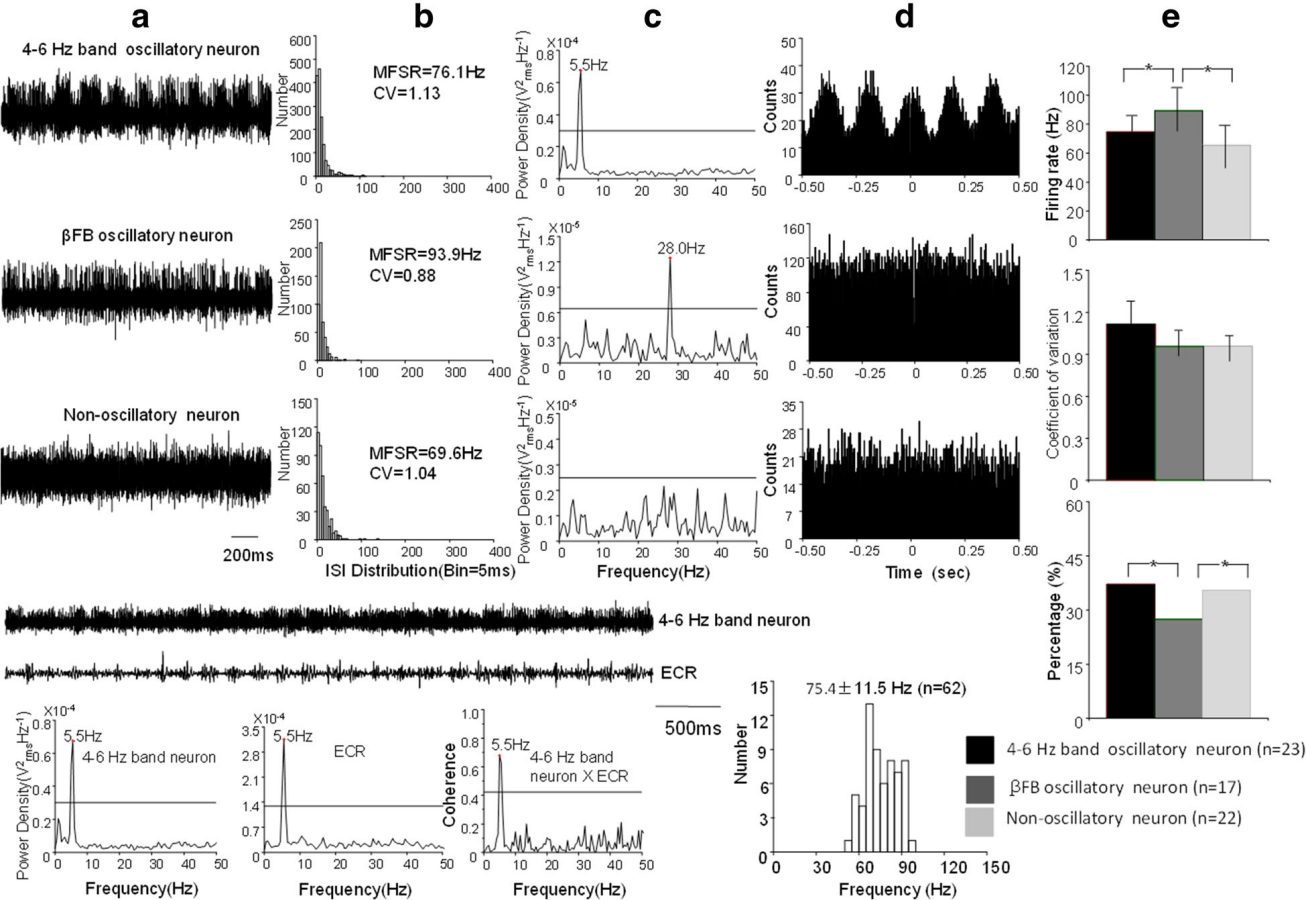
Characteristics of 4–6 Hz band, *β*FB, and non-oscillatory neurons in the GPi. **a** Patterns of three neurons with 4–6 Hz band, *β*FB, and non-oscillation. **b** ISI histograms of three neurons and their MSFR of 76.1 Hz, 93.9 Hz and 69.6 Hz. **c** Power density spectrum of the three neurons at peak power of 5.5 Hz, 28 Hz, and no power. **d** Autocorrelation histograms of three patterns of oscillatory neurons; **e** An example of GPi 4–6 Hz band oscillatory neurons and its coherence with limb tremor. The top trace shows raw data of a 4–6 Hz band oscillatory neuron that corresponds to the limb tremor; below shows spectral analysis of 4–6 Hz band oscillatory neuron, limb tremor of FCR, and 4–6 Hz band neuron x FCR, which is the coherence between them. Horizontal line indicates a significant coherent level at 0.42 (*p* < 0.05). **f** Comparisons of MSFR, CV and percentages of 4–6 Hz band, *β*FB oscillatory neurons and non-oscillatory neurons; G. Histogram demonstrates MSFR of total GPi neurons

Of 62 GPi neurons identified from 9 GPis, 23 (37.1%) neurons were 4–6 Hz band oscillatory neurons at frequency of 4.9 ± 0.6 Hz. Of these 4–6 Hz band oscillatory neurons, 6 (26.1%) were tremor related oscillatory neurons (coherent efficiency at range of 0.5–0.9, mean: 0.73 ± 0.14) (see fig. 2 E); (2) 17 (27.4%) were βFB oscillatory neurons with frequency of 15.7 ± 6.9 Hz (rang 10–28 Hz); (3) 22 (35.5%) were non-oscillatory neurons.

ISI analysis showed that the MSFR of 62 neurons were 75.4 ± 11.6 Hz (54.8–95.6 Hz) and of 40 oscillatory neurons was 80.9 ± 9.6 Hz (range 62.4–95.6 Hz) (*P <* 0.06).

Figure 2 illustrates examples of 4–6 Hz band and βFB oscillatory neuron and non-oscillatory neurons representative of most GPi neurons. The firing rate and CV and proportion of three patterns neurons were compared.

### Thalamic Vop and vim oscillatory neurons

Of 44 Vop neurons, 29 (65.9%) were 4–6 Hz band oscillatory neurons with peak frequency of 4.3 ± 0.4 Hz (range 4–5.5 Hz). Of these neurons, 14 (48.3%) were tremor related oscillatory neurons (coherent efficiency at range of 0.45–0.95, mean 0.75 ± 0.18); (2) 4 (9.1%) were βFB oscillatory neurons with frequency of 18.8 ± 7.2 Hz (range of 10–23 Hz); (3) 11 (25.0%) were non-oscillatory neurons. The autocorrelograms and ISI histograms demonstrate the three patterns of oscillatory and non-oscillatory neurons.

ISI analysis and histograms showed that the MSFR of 44 Vop oscillatory neurons were 23.4 ± 4.2 Hz (at range of 17.0–36.4 Hz) and of 33 oscillatory neurons was 24.5 ± 4.2 Hz (range 18.2–36.4 Hz) (*P* > 0.1).

Of 30 Vim neurons, 21 (70%) were 4–6 Hz band oscillatory neurons with frequency of 4.6 ± 0.7 Hz (range 4–6 Hz). Of these 4–6 Hz band oscillatory neurons, 11 (52.4%) were tremor related oscillatory neurons (coherent efficiency at range of 0.5–0.95, mean 0.7 ± 0.14); (2) 4 (13.3%) were βFB oscillatory neurons with frequency of 11.3 ± 4.9 Hz (range of 10–19 Hz); (3) 5 (16.7%) were non-oscillatory neurons.

ISI analysis showed that the MSFR of 30 neurons was 28.9 ± 4.7 Hz (range of 19.3–37.1 Hz) and of 25 oscillatory neurons was 30.3 ± 3.7 Hz (range of 22.9–37.1 Hz).

**Fig. 3:**
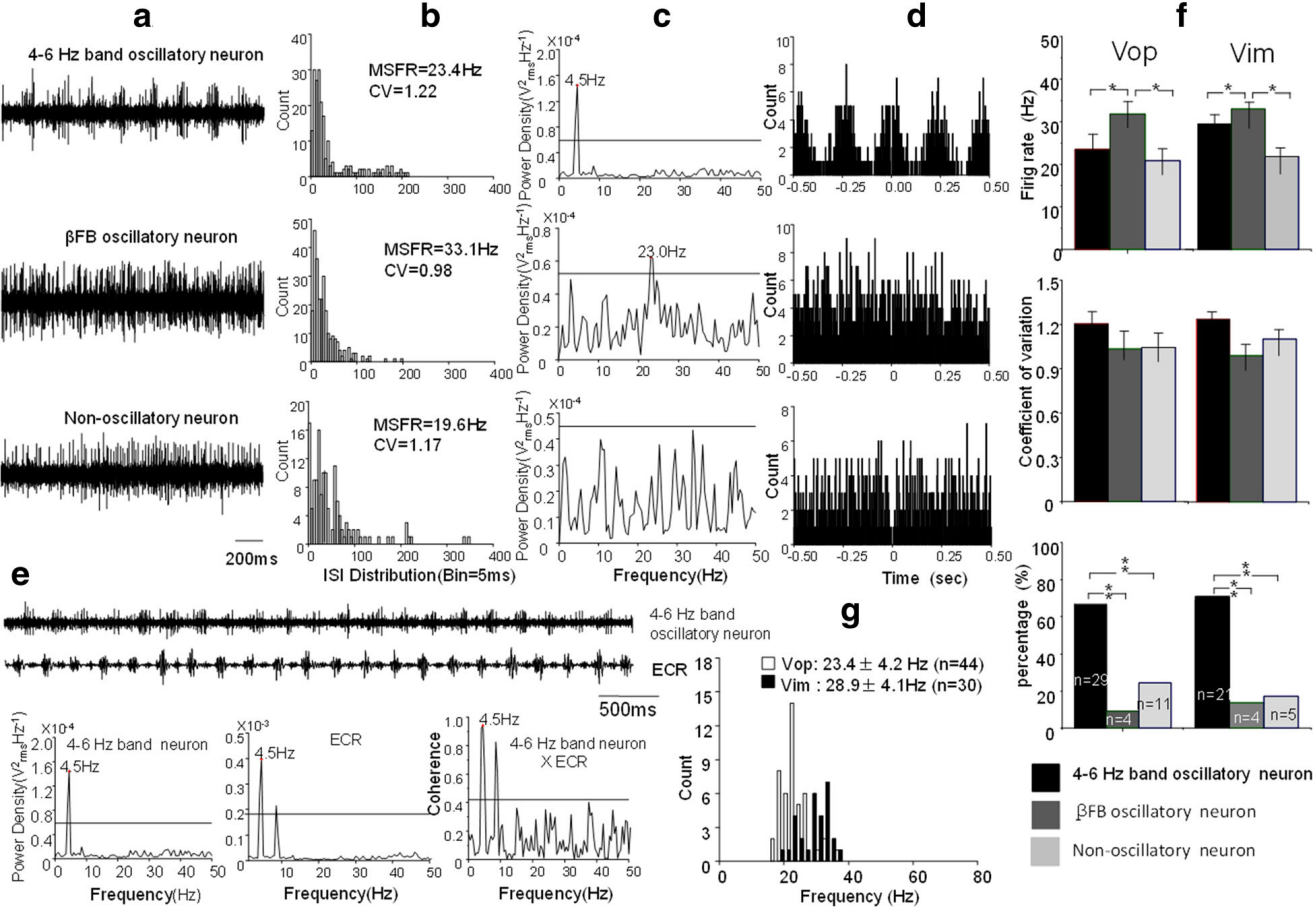
Characteristics of 4–6 Hz band, *β*FB, and non-oscillatory neurons in the Vop/Vim. **a** Patterns of three neurons with4–6 Hz band, *β*FB, and non-oscillation. **b** ISI histograms of three neurons and their MSFR of 23.4 Hz, 33.1 Hz and 19.6 Hz. **c** Power density spectrum of the three neurons at peak power of 4.5 Hz, 23 Hz, and no power. **d** Auto-correlation histograms of three patterns of oscillatory neuronal activity; **e** An example of Vop 4–6 Hz band oscillatory neurons and its coherence with limb tremor; The top trace shows raw data of a 4–6 Hz band oscillatory neuron that corresponds to the limb tremor (FCR); below shows spectral analysis of 4–6 Hz band neuron, limb tremor of FCR, and 4–6 Hz band neuron x FCR, which is the coherence between them. Horizontal line indicates a significant coherent level at 0.42 (*p* < 0.05). **f** Comparisons of MSFR, CV and percentages of 4–6 Hz band, *β*FB oscillatory neurons and non-oscillatory neurons of Vop and Vim. **g** Histograms demonstrate comparisons of MSFR of total neurons of Vop and Vim

Figure 3 demonstrated examples of 4–6 Hz band and βFB oscillatory neuron and non-oscillatory neurons representative for most thalamic neurons. The firing rate and CV and proportion of three patterns neurons were compared among the Vop neurons and Vim neurons.

### Comparison of the mean spontaneous firing rate of basal ganglia and thalamic oscillatory neurons

**Fig. 4:**
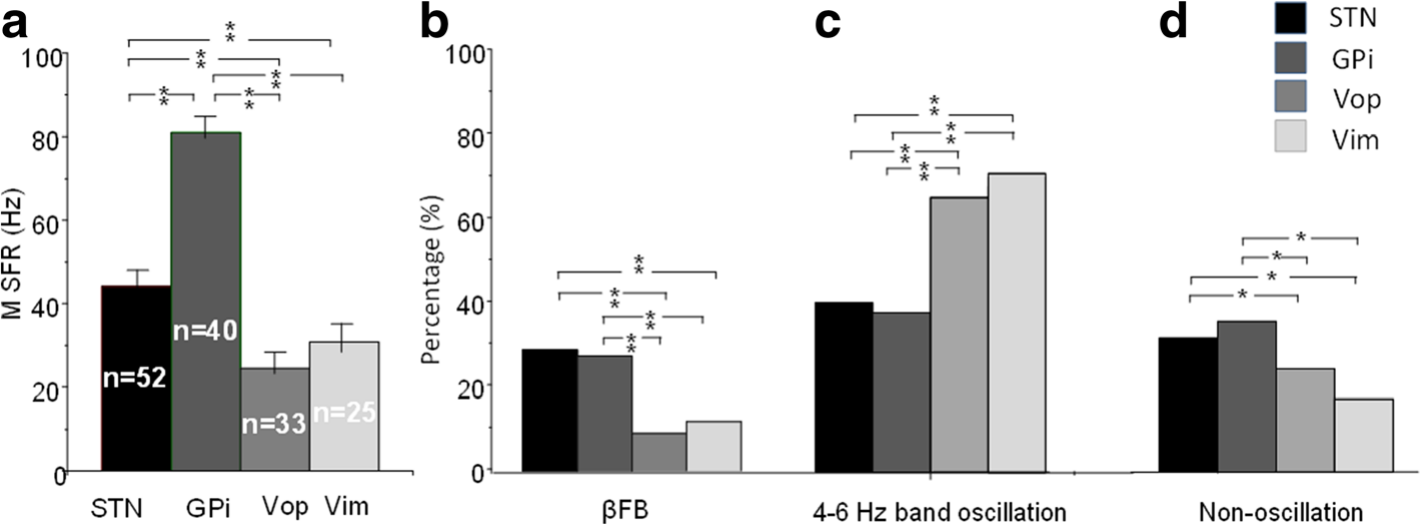
Comparison of the MSFR and percentage of oscillatory neurons in four nuclei. **a** Comparisons of MSFR of the oscillatory neurons of GPi, STN and Vop/Vim. ***p < 0.001; ^###^*p* < 0.001. **b** Comparisons of the percentage of two types of oscillatory neurons of STN, GPi, Vop/Vim. The percentage of *β*FB oscillatory neurons in GPi and STN were significantly higher than that of Vop and Vim; conversely, the percentage of 4–6 Hz band oscillatory neurons were significantly lower than that of Vop and Vim. *p < 0.05

MSFR of GPi oscillatory neurons was significant higher than that of STN oscillatory neurons (80.9 ± 9.6 Hz vs 44.2 ± 7.6 Hz) and this difference was statistically significant (*P* < 0.001) (see Fig. 4a).

MSFR of thalamic Vop oscillatory neurons was lower than that of Vim oscillatory neurons (24.4 ± 4.2 Hz vs. 30.3 ± 3.7 Hz) and the difference reached statistical significant (*P* < 0.01; see Fig. 4a).

Further ANOVA indicated there were significantly differences of MSFR of oscillatory neurons in STN, GPi and Vop/Vim (F = 460.7, df = 3, *P* < 0.0001). Bonferroni test indicated that all comparisons were significantly different (*P* < 0.001–0.05). Of four nuclei, the GPi neurons, the basal ganglia output neurons, showed highest neuronal firing rate whereas the Vop neurons, pallidal receiving neurons, demonstrated the lowest neuronal firing rate (80.9 ± 9.6 Hz vs 24.4 ± 4.2 Hz, *P* < 0.001).

Additionally, ANOVA showed that there were no significant differences of CV among three patterns of oscillatory neurons by four nuclei.

### Comparison of the proportion of basal ganglia and thalamic oscillatory neurons

Basal ganglia oscillatory neurons: a comparison of proportion of 4–6 Hz band oscillatory neurons and βFB oscillatory neurons by the STN and GPi was performed usin*g* X^*2*^ test. There was no significant difference of proportion of βFB oscillatory neurons (28.9% vs 27.4%, *P* > 0.05) and proportion of 4–6 Hz band oscillatory neurons (39.5% vs 37.1%; *P* > 0.05) between STN and GPi.

Thalamic oscillatory neurons: X^2^ test indicated that there was a marginal difference of proportion of Vop and Vim βFB oscillatory neurons (9.1% vs 13.3%) and proportion of Vop and Vim 4–6 Hz band oscillatory neurons (65.9% vs 70.0%, *P* = 0.06).

Further comparisons indicate that proportion of βFB oscillatory neurons in the STN and GPi were significant higher than that of similar oscillatory neurons in Vop and Vim (all *P* < 0.05, see Fig. 4b). In contrast, the proportion of 4–6 Hz band oscillatory neurons and tremor related oscillatory neurons in the Vop and Vim was significantly higher than that of similar neurons in STN and GPi (all *P* < 0.05, see Fig. 4c and Table 2); similar phenomena were also seen in non-oscillatory neurons (Fig. 4d).

**Table 2:** Analysis of 4–6 Hz band oscillatory neurons and tremor related oscillatory neurons in the basal ganglia nuclei and the Vop and Vim of thalamus

Patient	Oscillatory neurons (number)	4–6 Hz band oscillatory neurons (number)	Tremor related oscillatory neurons Number (percentage)	Range of coherent efficiency	Mean coherent efficiency (±SD)
STN (n = 11)	52	30	12/30 (40.0%)	0.45~ 0.85	0.68 ± 0.12
GPi (n = 9)	40	23	6/23 (26.1%)	0.5~ 0.9	0.73 ± 0.14
Vop/Vim (n = 9)	33/25	29/21	14/29 (48.3%)*,^#^ /11/21 (52.4%)^*^,^#^	0.45~ 0.95 /0.55~ 0.95	0.75 ± 0.18 /0.82 ± 0.14

STN: subthalamic nucleus, GPi: globus pallidus internus; Vop: ventral oral posterior of thalamus; Vim: ventral intermediate of thalamus; *: STN tremor related oscillatory neurons: compared to Vim, and Vop, *p* < 0.05; ^#^ GPi compared to Vop and Vim, *p* < 0.05; ^&^: STN compared to Vim, *p* < 0.05

Table 2 further demonstrates comparisons of tremor related oscillatory neurons in the STN, GPi and Vop/Vim. There were significantly different proportions of tremor related oscillatory neurons between STN, GPi and thalamic Vop/Vim (all *P* < 0.05). Furthermore, there was also a significant difference of mean coherent efficiency reached between STN and Vim tremor related neurons (0.68 vs 0.82, *P* < 0.05).

## Discussion

The current study characterized properties of oscillatory neurons in the basal ganglia and thalamus in patients with PD. Consistent with previous results [5, 8–11, 17, 19, 23, 26], we confirm and extend the previous findings that alterations in firing rate and pattern of oscillatory neurons in the basal ganglia and thalamus are associated with parkinsonian symptoms. In addition to 4–6 Hz band and βFB oscillatory neurons identified in basal ganglia nuclei STN, GPi and the Vop/Vim of thalamus, there are three novel findings: First, of four nuclei, the highest MSFR is in the GPi oscillatory neurons (80.9 ± 9.6 Hz), the basal ganglia output neuron, whereas the lowest MSFR is Vop oscillatory neuron (24.4 ± 4.2 Hz), the presumed pallidal receiving area of thalamus. The data do match the prediction that hyperactivity of the basal ganglia output structures results in a reduction of thalamic activity [3, 4]. Second, the proportion of βFB oscillatory neurons is more prominent in the basal ganglia nuclei than in the Vop/Vim of thalamus. βFB oscillatory neurons have been thought to be antikinetic, suggesting that βFB oscillatory neuron is likely associated with dopamine depletion in the basal ganglia. The third, both basal ganglia STN, GPi and thalamic Vop/Vim have 4–6 Hz band and tremor related oscillatory neurons, however, the proportion of Vop/Vim 4–6 Hz band oscillatory neurons and tremor related oscillatory neurons is higher than that of STN and GPi suggesting that while both basal ganglia and thalamus are involved in generation of tremor, the thalamus likely plays a more important role in tremor production [44, 45]. However, the basal ganglia β oscillatory activity might be a trigger. Stimulating and abolishing the abnormal activities results in improvement of parkinsonian symptoms. These findings indicate the critical role of abnormal neuronal activity in the basal ganglia-thalamocortical circuit in the generation of PD symptoms. The results presented here are generally in support of the “classic” model of basal ganglia dysfunction in PD [3, 4].

In the present study, patients with different phenotypes are selected different targets for the treatment. Since the indications differed for the different targets, some of the different physiological results in the targets might be due to different clinical features. In order to exclude possible clinical features of the patients that might affect neuronal activity, we recorded a sufficient number of neurons in patients with different subtypes of PD, under sufficiently similar conditions, including both awake and anesthetized states. In addition, we also systematically analyzed the data according to the current medication status. However, we did not detect evidence for an effect of any of these drugs on neuronal firing.

Alterations in neuronal activity in the basal ganglia-thalamocortical “motor” circuit have been proposed to explain many clinical features associated with the model of hypokinetic and hyperkinetic movement disorders, and the former has been taken into account for parkinsonian motor deficits [3, 4]. The present finding of increased firing rate of GPi oscillatory neurons and decreased firing rate of Vop oscillatory neurons fit with the “rate model” of PD. The model has been proposed to link the development of the hypokinetic features of PD. According to the model, increased pallidal (inhibitory) output results in decreased firing rate in the pallidal receiving area of thalamus, leading to parkinsonian motor symptoms [3, 4, 46]. In the MPTP treated parkinsonism monkey and patients with PD, microelectrode recording studies suggested that increased firing rates and bursting activity in the STN and GPi account for the hypokinetic features of PD [4, 46]. Soares et al. [9] demonstrated that MPTP induced an increased in the average discharge rate of STN neurons from a control value of 25.7 Hz to 36.1 Hz, and in GPi from 65.1 Hz to 80.6 Hz in a primate study. Similarly, Heimer and his colleagues demonstrated that the MSFR of GPi neurons increased from 62.9 Hz to 75.3 Hz after dopamine depletion in a parkinsonian primate study [7]. Thus, our findings of MSFR of STN (44.2 Hz) and MSFR of GPi neurons (80.9 Hz) in PD patients are compatible with these findings of animal studies. Conversely, decreased firing rate has been found in monkeys’ VLo (the human equivalent of Voa/Vop) in MPTP-treated versus normal animals [1, 18], and MSFR of Voa/Vop of thalamus in PD patients who underwent surgery as compared to patients with essential tremor and pain [16, 17]. In the current study, we found that MSFR of Vop (24.4 Hz) in patients with PD was significant lower than MSFR of GPi (80.9 Hz). These findings do fit the prediction that hyperactivity of the basal ganglia output structures results in a reduction of thalamic activity [3, 4]. In the current study, our finding of MSFR of Vop neurons (24.4 Hz) was higher than that of Vop neurons (7.4 Hz) in Molnar’s study [16]. One explanation might be that we calculated MSFR for Vop neurons during the rest condition and Vop neurons were defined by location > 3 mm from tactile border of Vc. Molnar’s group calculated MSFR was for Vop neurons that were voluntary or passive movement related or by location > 2 mm or < 2 mm from the tactile border [16].

A number of studies have focused on the role of βFB oscillatory neurons in relation to bradykinesia and rigidity either in LFP studies [47] or microelectrode recordings studies [23, 48, 49]. There is wide agreement on the association of β activity in cortico-basal ganglia loops (13-30 Hz) with static motor control, such as tonic or postural contraction [49]. In PD, in the absence of levodopa medication, the cortico-basal ganglia loop tends to synchronize within the β band. However, after levodopa replacement treatment, they tend to synchronize with higher frequency (> 70 Hz) [28, 50, 51]. DBS at 20 Hz (β frequency band) of the STN synchronized GPi at the same frequency whereas high frequency (> 70 Hz) STN DBS suppressed β frequency GPi oscillations. Consistent with direct modulation of these oscillations having clinical effects, STN and GPi high frequency stimulation improves PD motor symptoms, while β frequency stimulation of STN has an antikinetic effect in PD patients [50]. Levodopa administration, which is effective treatment of bradykinesia, decreases basal ganglia β oscillation [38, 39]. Moreover, a recent study demonstrated that STN β oscillatory neurons directly correlate with limb muscular activity of PD patients with akinetic-rigid type [30]. The proportion of β oscillatory neurons was higher in STN in the akinetic-rigid type than that of β oscillatory neurons in mixed type [30, 40]. Furthermore, cortical β oscillations are inversely correlated with movement acceleration. Thus, it can be argued that enhanced basal ganglia β band activity could be considered as an indicator of bradykinesia, modulated by dopaminergic agents [51–53]. The present results demonstrate prominent β oscillatory neurons in the STN and GPi whereas there are less β oscillatory neurons in the Vop/Vim. With this regard, the exaggerated β activity is likely to be most prominent in the parkinsonian basal ganglia [51–53].

Several lines of studies focused on the role of basal ganglia and thalamus in generation of tremor. Tremor frequency activity has been observed in GPi [21, 24, 26] and STN [23, 26, 54] and Vim (posterior VL) of thalamus [17, 18, 28, 55] during intra-operative recordings in PD patients undergoing surgery. Hutchison and colleagues [24] clearly demonstrated a linear relationship between the peak frequency of limb tremor and GPi tremor frequency activity suggesting that GPi is involved in the genesis of rest tremor. They concluded that the findings are in support of the hypothesis of Albin [3] and DeLong [4] that excessive neuronal activity in GPi promotes tremorgenesis. Hurtado and his colleague demonstrated that there was a dynamic relationship between GPi neuronal activity and limb tremor suggesting the existence of transiently synchronized limb-GPi oscillations and independent tremor-related activity in GPi [26]. Furthermore, intra-operative recording of LFPs in the STN of patients with tremor dominant PD revealed clusters of tremor-associated coupling between STN and tremor EMG [56]. Another study using intra-operative STN recordings in patients with tremor found that neurons (episodically) oscillating at tremor frequency were locally surrounded by non-oscillating or out-of-phase neurons, while large populations of neurons continuously oscillated at 8–20 Hz [23]. Our previous study using microelectrode recordings in STN in patients with PD found that 4–6 Hz band oscillatory neurons were significantly coherent with limb tremor EMG. These tremor frequency neurons are intermixed with neurons firing at β frequency. The finding suggests involvement of basal ganglia [26, 44]. Lenz’s group had a series of studies on the role of the ventral nuclear group of thalamus in the pathogenesis of tremor. They demonstrated that the thalamic “tremor cells” in VL were correlated with “3–6 Hz” component of EMG of parkinsonian tremor [25] and these tremor cells often have a phase lead relative to EMG activity during tremor [55]. They further found that the majority of these tremor frequency cells were located in Vim and Vop and these Vim and Vop cells were not significantly different in 4–6 Hz band oscillatory activity [55, 57]. Consistent with these findings, we found a similar proportion of 4–6 Hz band oscillatory neurons and tremor related oscillatory neurons localized in Vop (65.9%; 48.3%) and Vim (70.0%; 52.4%) during tremor. The nuclei were also not significantly different either in the proportion of 4–6 Hz band oscillatory neurons or of tremor related oscillatory neurons. The similarities between neuronal activities in Vop and Vim of thalamus are striking given the differences in their connections and physiology. These similarities might result from errors in the radiological determination of nuclear boundaries so that the nuclear location of cells was not accurate identified. Alternately, PD might have altered the activity of cells in Vim, Vop or both, since there are interactions between basal ganglia and cerebellum [45, 46, 58]. The present results together with previous findings suggest that parkinsonian tremor may result from a pathological interaction between basal ganglia circuits and cerebellothalamic circuits. The basal ganglia circuits may trigger the onset of tremor and cerebellothalamic circuits may be responsible for the amplitude [44, 45, 58]. Consistent with this, we also demonstrated that the highest mean coherence coefficient of tremor related oscillatory neurons has been observed in Vim, although the finding needs to be further confirmed.

In this study, patients with prominent tremor were selected for thalamotomy. The highest proportion of tremor frequency oscillatory neurons and the highest mean coherence coefficient of tremor related oscillatory neurons were observed in the Vop/Vim in these patients. A limitation of the study is that we do not have data on recordings in tremor predominant patients in the STN or GPi. The result relating to the proportion of tremor neurons may then be biased. On the other hand, the high coherence strongly supports the view that the thalamus is more important in the generation of tremor.

One major shortcoming of our study was the small sample size of 29 patients, which decreased the *p* value of our statistical results. Another limitation of this study was that we collected pre-surgery UPDRS III data but not post-surgery UPDRS III data, so clinical outcome of patient were not evaluated. We expect that future studies with larger patient populations will confirm these findings.

## Conclusions

The current findings support the hypothesis that alteration in the neuronal activity in the basal ganglia structures plays a critical role in generation of parkinsonian motor deficits [3, 4, 45]. β oscillatory activity predominantly exists in the PD basal ganglia supporting the view that the activity results from dopaminergic depletion. With parkinsonian tremor, there appears to be dysfunction of the basal ganglia and the cerebello-thalamo-cortical network, but the latter seems to be more important [44, 58].

## Abbreviations

CRW: Cosman-Roberts-Wells
CV: Coefficient Variation
ECR: The extensor carpi radialis
EMG: Electromyography
FCR: The flexor carpi radialis
GPe: The external segment of globus pallidus
GPi: The internal segment of globus pallidus
ISI: Interspike interval
LFP: The local field potential
MPTP: 1-methyl-4-phenyl-1,2,3,6-tetrahydropyridine
MSFR: Mean spontaneous firing rate
PD: Parkinson’s disease
SNr: The substantial nigra reticularis
STN: Subthalamic nucleus
TFB: Tremor frequency band
THE UPDRS: The Unified Parkinson’s Disease Rating Scale
VC: The ventral caudal of thalamus
VIM: The ventral intermediate of thalamus
VOA: The ventral oral anterior of thalamus
VOP: The ventral oral posterior of thalamus
βFB: β frequency band
